# Research on fault identification of high-voltage circuit breakers with characteristics of voiceprint information

**DOI:** 10.1038/s41598-024-59999-0

**Published:** 2024-04-23

**Authors:** Sihao Wang, Yongrong Zhou, Zhaoxing Ma

**Affiliations:** 1 State Grid Electric Power Research Institute Co., Ltd., Nanjing, 211106 China; 2 School of Information and Control Engineering, Qingdao University of Technology, Qingdao, 266520 China

**Keywords:** Energy science and technology, Engineering, Electrical and electronic engineering, Mechanical engineering

## Abstract

High voltage circuit breakers are one of the core equipment in power system operation, and the voiceprint signals generated during operation contain extremely rich information. This paper proposes a fault identification method for high voltage circuit breakers based on voiceprint information data. Firstly, based on the developed voiceprint information data acquisition device, the voiceprint information of a certain high voltage circuit breaker is obtained; Secondly, an improved S-transform is proposed in the article, which generates an amplitude matrix based on the S-transform of voiceprint information; Then, through the matrix Singular value decomposition method, the fault feature quantity of voiceprint information is extracted from the time–frequency angle, and the diagnosis system of the support vector machine model is established, and the system is trained to realize the fault identification of the high-voltage circuit breaker; Finally, through experimental simulation calculations, it was shown that the accuracy of the proposed fault identification method in different operating conditions reached 92.6%, verifying the good accuracy and robustness of the proposed method and equipment.

## Introduction

High-voltage circuit breakers are crucial equipment in the power system, and their operating conditions have a significant impact on the entire system, sometimes even affecting the safe and reliable operation of the entire network system^1,2^. Therefore, monitoring and fault identification of circuit breakers is a very important task, where circuit breaker faults generally include mechanical and non-mechanical faults. Statistics from the International Conference on Large-Scale Power Systems indicate that mechanical faults are the main type of fault, accounting for approximately 80%^3^. By acquiring the voiceprint information generated during the operation of the circuit breaker and applying fault feature extraction methods, effective monitoring of the operation status of the circuit breaker is an important means to achieve fault diagnosis of high-voltage circuit breakers. This article will conduct research in this area.

There are currently two main technical routes for the diagnosis of high-voltage circuit breaker faults: vibration signal-based and voiceprint information-based. Although there are similarities between the two routes, both require obtaining information, extracting features, and identifying features through algorithms. However, the research techniques and methods are very different, including the equipment required to collect signals, which also varies greatly. In recent years, many researchers have proposed ideas and methods for applying vibration signals of circuit breakers to achieve fault diagnosis^4–6^. There have been some achievements in using voiceprint information to detect faults in power systems^7^, but there are few research results on using voiceprint information to identify high-voltage circuit breaker faults. High-voltage circuit breakers contain many components with complex structures, and there is a coupling relationship between the components during the action process. Using vibration signals to diagnose faults requires high complexity in signal acquisition equipment and high requirements for the installation location and installation method of sensors. If the installation location is not selected properly, it will have a significant impact on the results, resulting in poor universality. Another technical method is to identify mechanical faults in high-voltage circuit breakers based on voiceprint information. Although it is homologous to vibration signals, it has its own advantages. The voiceprint information acquisition equipment is low in complexity, high in reliability, easy to install and use in substations, and easy to maintain, making it highly scalable. At the same time, it has high accuracy in fault identification, and its key and difficult points are mainly reflected in the acquisition, feature extraction, and classification identification of voiceprint information. This article will also study the acquisition of voiceprint information, feature extraction methods, and classification identification algorithms for high-voltage circuit breakers.

At present, many researchers have achieved many effective results in fault diagnosis of high-voltage circuit breakers. Research shows that circuit breaker fault diagnosis generally involves three main aspects: data acquisition, feature extraction, and fault diagnosis identification. In terms of time–frequency transformation, the main methods applied include Fourier transform^8^, wavelet transform^9^, chirp-z transform^10^, Hilbert transform^11^, S transform^12,13^, and so on. In the application of Fourier transform, as a global transformation method, Fourier transform lacks the function of time and frequency localization, and is generally suitable for analyzing stationary signals. As a windowed Fourier transform method, the length of the window function determines the temporal and frequency resolution of the signal. In addition, due to the limitations of Heisenberg's uncertainty criterion, it is difficult to obtain a good time-domain distribution and frequency-domain distribution simultaneously when using Fourier transform for time–frequency analysis of signals. Wavelet transform, as a time–frequency analysis method with variable window function, overcomes the shortcomings of Fourier transform and has the characteristic of multi-resolution analysis. However, the selection of wavelet basis functions lacks adaptability, and when using wavelet transform to analyze signals, there are shortcomings such as frequency interference, energy leakage, and boundary distortion in the time–frequency spectrum. The Chirp-z transform has improved frequency resolution compared to the Fourier transform, but it produces more low-frequency harmonics, which has a certain impact on accuracy; The Hilbert transform has high efficiency in extracting transient signals, but there are limitations on the transformation conditions. The S-transform was first proposed by Stockwell^12^, who drew on the ideas of Fourier transform and wavelet transform, and had its own advantages. The S-transform uses a variable Gaussian window function, whose width is inversely proportional to frequency, resulting in better time–frequency characteristics.

The calculation and analysis methods for fault diagnosis of high-voltage circuit breakers mainly include support vector machines^14,15^, wavelet analysis^9^, short-term energy analysis^16^, empirical mode decomposition^17^, information entropy^18^, transfer learning approach^19^, etc. Compared to other methods, the support vector machine method has significant advantages in dealing with small sample and nonlinear problems. It is very difficult to obtain data from high-voltage circuit breaker voiceprint monitoring, and the collected voiceprint information data has the characteristic of small sample. The support vector machine method has high potential for application in fault identification of high-voltage circuit breaker voiceprint monitoring in small sample scenarios.

This article conducts fault diagnosis research on voiceprint monitoring of high-voltage circuit breakers under different working conditions, develops a voiceprint monitoring device, and builds an experimental system to simulate different types of faults. The rest of the organization of this manuscript is as follows: Section “High voltage circuit breaker voiceprint information data acquisition device” explains the design and development process of the voiceprint information acquisition device. Section “Fault feature extraction methods and application analysis” provides a comprehensive method for extracting fault features using voiceprint information, including fault diagnosis. Section “Experiments and analysis” presents the analysis results of the experimental system for fault diagnosis of high-voltage circuit breakers using voiceprint monitoring information. Finally, concluding remarks are mentioned in Section “Conclusions”.

## High voltage circuit breaker voiceprint information data acquisition device

### Hardware design

The voiceprint monitoring device mainly consists of an MSP chip, a program download interface on its left side, a chip reset circuit, three dial switches, and an external crystal oscillator circuit. Among them, in the slave sampling circuit, due to the positive power supply of the audio part's operational amplifier and the positive and negative power supply of the magnetic field part's operational amplifier, the power module of the slave is divided into two parts. One part generates a positive power supply through the MOS transistor CJ3401 and the voltage regulator BL8565 and its peripheral circuits, and the other part generates a positive and negative power supply through the voltage regulator LM27762 and its peripheral circuits.

The voiceprint sampling circuit consists of three reference voltage chips, a programmable amplification instrument amplifier AD8231ACPZ, and an operational amplifier OPA365AIDR. The principle is to convert the sound signal into an analog voltage signal that fluctuates up and down at 1.2 V through the vibration of the eardrum of the acoustic sensor (microphone sensor), and then amplify the differential voltage signal through the programmable operational amplifier AD8231ACPZ. Finally, the differential voltage signal is input to the main control chip after being reversed by the following amplifier OPA365AIDR.

### Data storage card design

**Figure 1:**
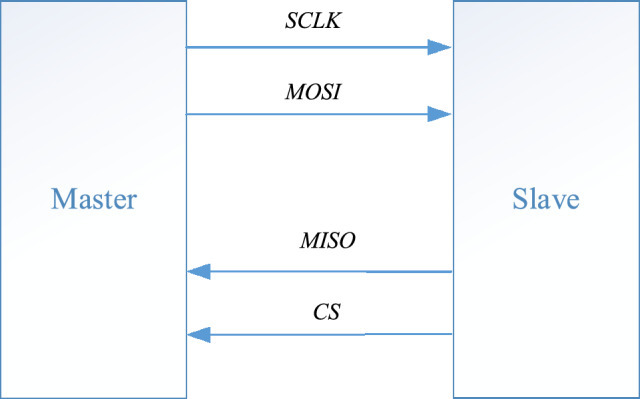
Master slave connection method.

The SD card adopts a serial peripheral protocol working mode, and the serial peripheral protocol interface is also known as the serial peripheral interface. The serial peripheral protocol interface operates in a master–slave mode, and the master–slave connection method of the SD card is shown in Fig. 1.

This mode usually has one main device and one or more slave devices, and its interface includes the following four types of signals:

1. MOSI—Master device data output, slave device data input;
2. MISO—Main device data input, secondary device data output;
3. SCLK—Clock signal, controlled by the main device;
4. CS—Enable signal, controlled by the main device.

### Device power supply design

Considering the application scenarios of the device, there are two methods for power supply: 1 External maintenance power supply method; 2. Built in lithium battery mode. The first scenario is suitable for continuous monitoring of a circuit breaker in intensive care (in days). The second scenario is suitable for short-term diagnostic analysis of multiple circuit breakers.

### Device integration design

**Figure 2:**
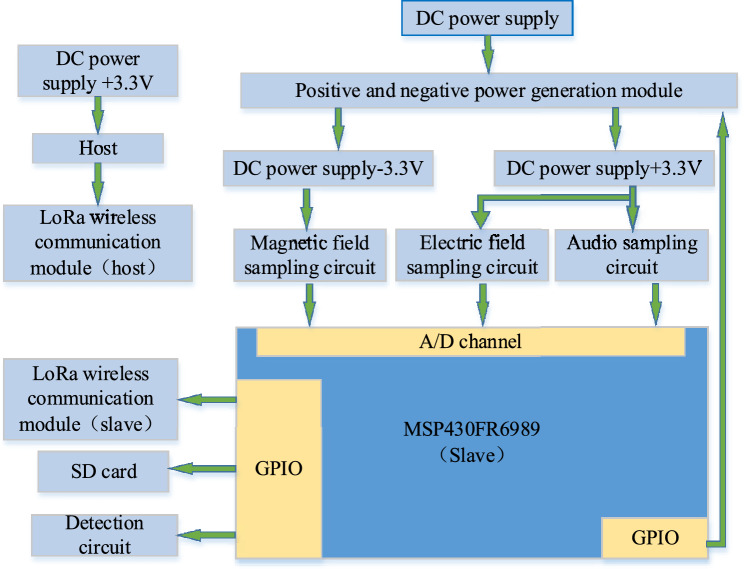
Structure diagram.

The online voiceprint monitoring device for the opening and closing status of high-voltage circuit breakers uses TI's MSP430FR6989 chip to process the sampling signal. As shown in Fig. 2, the system structure diagram mainly includes MSP and its peripheral circuits, sampling circuit, positive and negative power generation module, detection circuit, LoRa wireless communication module, etc.

**Figure 3:**
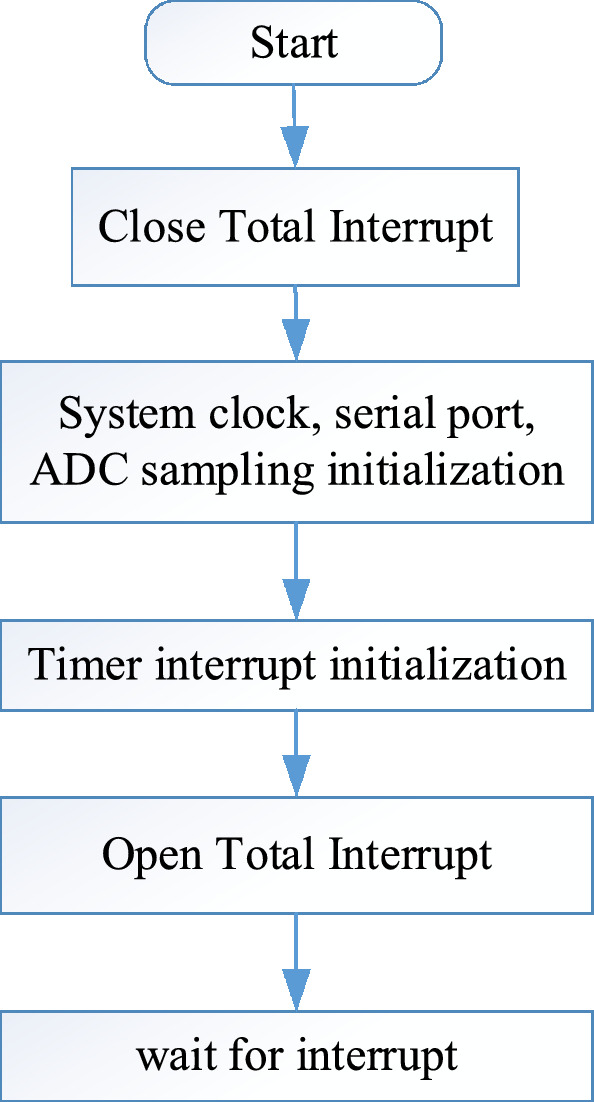
Slave master program flowchart.

**Figure 4:**
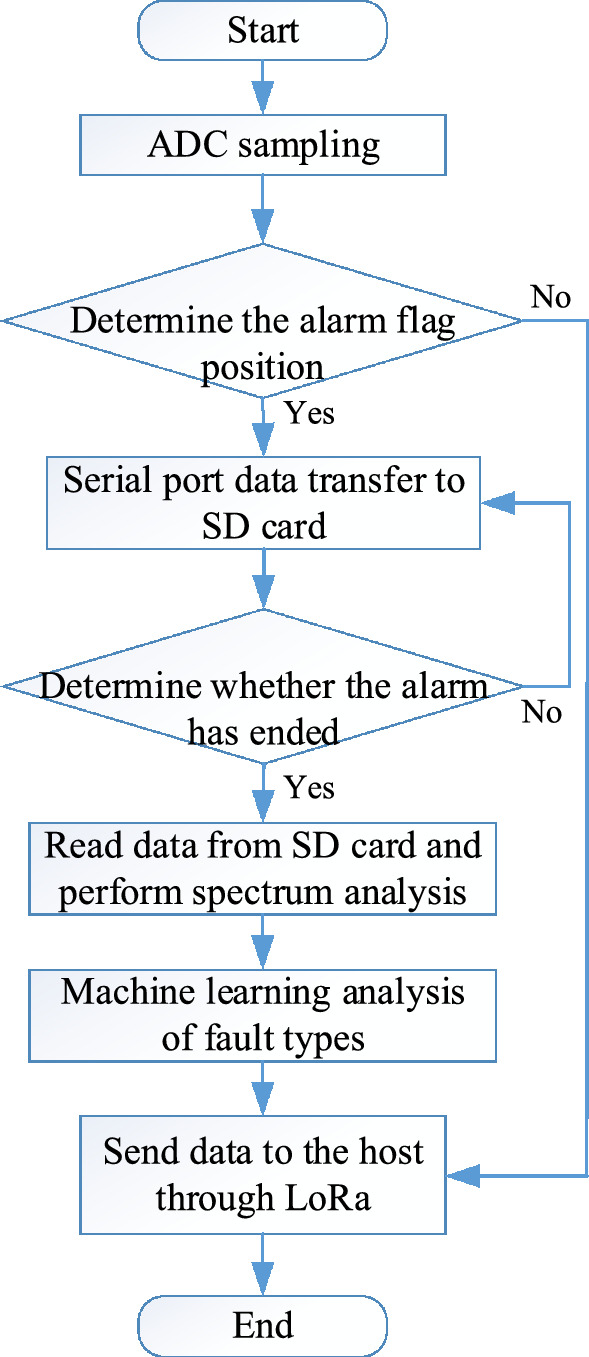
Slave interrupt subroutine flowchart.

The main functions of the slave sampling program designed and written based on the MSP430FR6989 chip are initialization of various MSP variables, ADC sampling, timer interruption, entering the main loop, SD card data reading and writing, spectrum analysis, calculation, etc. The main program flowchart of the slave software design is shown in Fig. 3, and the interrupt subroutine flowchart is shown in Fig. 4.

**Figure 5:**
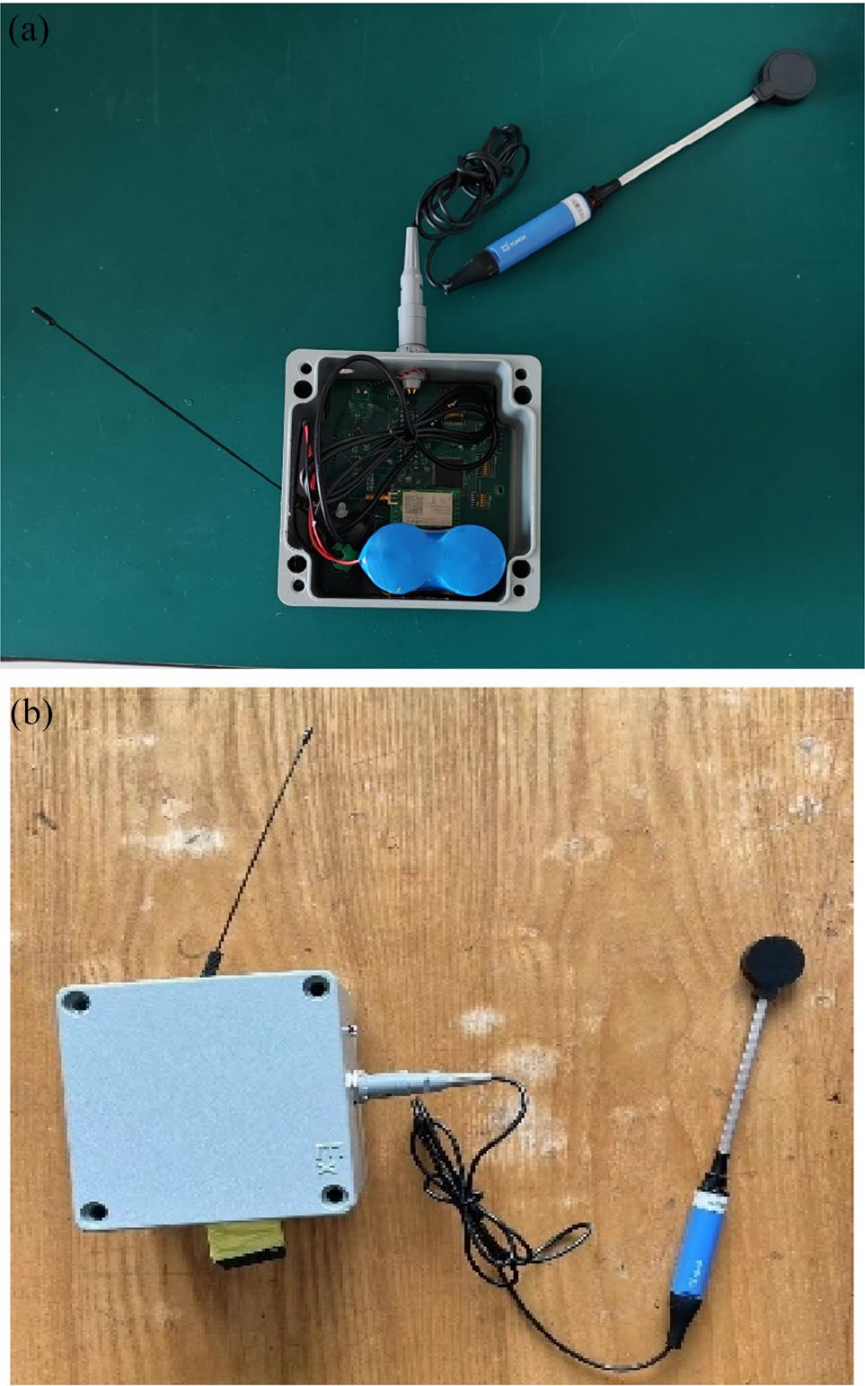
Voiceprint monitoring device. (**a**) Internal circuit of voiceprint monitoring device. (**b**) Appearance of voiceprint monitoring device.

The internal circuit and appearance of the device after development are shown in Fig. 5.

## Fault feature extraction methods and application analysis

### Improved S-transformation and establishment of feature matrix

The circuit breaker voiceprint information obtained in this study is a one-dimensional time-domain signal, and to extract its inherent features, time–frequency transformation of the voiceprint information is necessary. Generally, the transformation methods for studying time–frequency information include Fourier transform, wavelet transform, Hilbert transform, etc. Different transformation methods have different characteristics and are applicable to different scenarios. This article adopts an improved S-transform method to perform time–frequency transformation on voiceprint information.

Let the continuous time-domain signal be *y* (*t*), and its S-transform is represented as^12,13^:

1 $$ S\left( {\delta ,f} \right) = \int_{ - \infty }^{ + \infty } {y\left( t \right)\varphi \left( {\delta - t,f} \right)e^{ - 2j\pi t} dt} $$

2 $$ \varphi \left( {t,f} \right) = \frac{\left| f \right|}{{\sqrt {2\pi } }}e^{{ - \frac{{f^{2} t^{2} }}{2}}} $$

where *f* is the frequency, *φ*(*δ*-*t*,* f*) is a Gaussian window function, *δ* is a control parameter for the position of the timeline.

From Eq. (2), it can be observed that *φ*(*δ − t*,* f*) function is a continuous function of time and frequency, with a smooth variation relationship with frequency. Its width is inversely proportional to frequency and time, providing higher frequency resolution at lower frequencies and higher time resolution at higher frequencies. Now, for the window function of Eq. (2), take the partial derivative about frequency (the frequency of the voiceprint information data of the circuit breaker studied in the article is all positive), expressed as follows:

3 $$ \frac{\partial \varphi }{{\partial f}} = \frac{1}{{\sqrt {2\pi } }}e^{{ - \frac{{f^{2} t^{2} }}{2}}} \left( {1 - f^{2} t^{2} } \right) $$

The duration of the opening and closing sound pattern information of the circuit breaker is extremely short, at the millisecond level, and the frequency is at the kHz level. Therefore, the value of Eq. (3) is very small, close to zero, or even equal to zero. Therefore, in practical applications, when the frequency changes within a certain range, the S transformation will have insufficient energy aggregation and resolution. To address this deficiency, this article proposes an improved S transformation method, which introduces a parameter *k* to the window function of Eq. (2). The new window function is represented as follows:

4 $$ \varphi^{\prime}\left( {t,f} \right) = \frac{{\left| {kf} \right|}}{{\sqrt {2\pi } }}e^{{ - \frac{{k^{2} f^{2} t^{2} }}{2}}} $$

In Eq. (4), when *k* = 1, it is the same as the window function in Eq. (2). Therefore, by changing different *k* values, the width of the window function can be adjusted. In this paper, *k* = 10 is taken. In the article, for the voiceprint information of circuit breakers, discrete time variables are collected, and the S transformation given in Eq. (1) is for continuous variables. Therefore, for the convenience of engineering application, it is necessary to change Eq. (1) and provide the S transformation expression for the discrete time series *y*(*n*), as shown below:

5 $$ S\left( {it_{0} ,\frac{n}{{Nt_{0} }}} \right) = \sum\limits_{m = 0}^{N - 1} {y^{\prime}\left( {\frac{n + m}{{Nt_{0} }}} \right)e^{{ - \frac{{2\pi^{2} m^{2} }}{{k^{2} n^{2} }}}} } e^{{\frac{j2\pi mi}{N}}} ,\;\;\;\;\;\;\;n = {1},{2}, \ldots ,N - {1} $$

6 $$ S\left( {it_{0} ,0} \right) = \frac{1}{N}\sum\limits_{m = 0}^{N - 1} {y^{\prime}\left( {\frac{m}{{Nt_{0} }}} \right)} ,n = 0 $$

where $y^{\prime}\left( {\frac{n}{{Nt_{0} }}} \right)$ is the discrete Fourier transform of *y*(*n*), *t*_0_ represents the data acquisition interval and *N* represents the total number of points used.

According to the theoretical methods of Eqs. (5) and (6), and S transformation, the transformation result is a complex matrix, with rows representing different frequency values, columns representing corresponding time points, and matrix elements representing the amplitude and phase angle of the signal. Let the complex matrix of the obtained transformation result be *S*_*l*×*n*_, and the S transformation can be represented by the following equation^20^:

7 $$ S_{l \times n} = T_{l \times n} e^{{j\theta_{l \times n} }} $$

where $T_{l \times n}$ is the amplitude matrix, $\theta_{l \times n}$ is the phase matrix.

### Method for extracting fault features

Regarding the method of fault feature extraction, this section adopts the mathematical idea of combining singular value decomposition and support vector machine. For matrices, singular values can characterize their inherent characteristics, have excellent stability, contain rich information^21^, and are widely used in pattern recognition, especially in extracting feature information from time–frequency matrices^22^.

For amplitude matrix *B*_*l*×*n*_, orthogonalization decomposition of the matrix can be performed, therefore, there must be two matrices *L* and *N* that satisfy the following relationship:

8 $$ B = LCN $$

9 $$ C = [diag\left( {c_{1} ,c_{2} , \cdots ,c_{p} } \right),0] $$

where *L* is *l* × *l* matrix, *N* is *n* × *n* matrix, *C* is a diagonal matrix, *C* ∈ *R*^*l*×*n*^; 0 is a zero matrix, *c*_*i*_ is the singular value of matrix *B*, and *p* is the rank of matrix *B*.

According to the partitioning of the matrix, matrix *B* can also be represented by the following equation:

10 $$ B = \sum\limits_{j = 1}^{p} {c_{j} B_{j} } $$

where *B*_*j*_ is a partitioned matrix of matrix *B.*

Therefore, the features of the original data will be mapped onto the set of feature vectors, so that the data can still retain the feature information of the original data even in the case of dimensionality reduction. To better reflect the features of the original data after dimensionality reduction, the feature vector matrix is analyzed and processed. The feature matrix is multiplied by the sum centrality matrix (subtracting the mean of each one-dimensional feature from the original data to obtain a new matrix, which is called the centrality matrix). The new feature matrix is input into the support vector machine model for training and classification, The classification calculation method is described below.

### Support vector machine classification method

When using feature identification, this article adopts the idea of support vector machine, inputting the feature vector composed of singular values into the support vector machine model, and achieving the purpose of fault identification through training.

**Figure 6:**
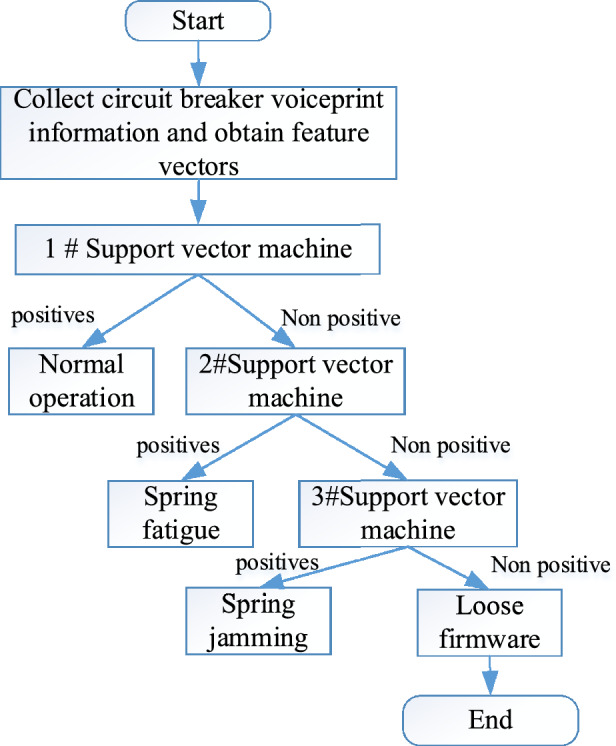
1-v-1 support vector machine identification process.

At first, support vector machines were generally used for classification of Class 2 problems, but in practical applications, the problems were relatively complex and there were many classifications. Therefore, many improved support vector machine methods have emerged^23,24^. During the operation of high-voltage circuit breakers, they may operate normally or other types of faults may occur, such as normal operation, spring fatigue, spring jamming, firmware loosening, etc. The 1-v-1 support vector machine method classifies multiple operating conditions. Generally, one support vector machine can classify two types of conditions. The training and identification process is shown in Fig. 6.

As shown in Fig. 6, the obtained voiceprint feature vector is used as input to the 1# support vector machine. Positive samples are used to identify normal operating conditions, and then non positive samples are input to the 2# support vector machine. Positive samples identify spring fatigue, and non positive samples are further input to the 3# support vector machine. Positive samples identify spring jamming, while non positive samples indicate firmware loosening. From the calculation idea in Fig. 6, it can be seen that the 1-v-1 support vector machine method consumes higher computational costs and has lower computational efficiency. Therefore, it is necessary to seek suitable methods for multi classification.

**Figure 7:**
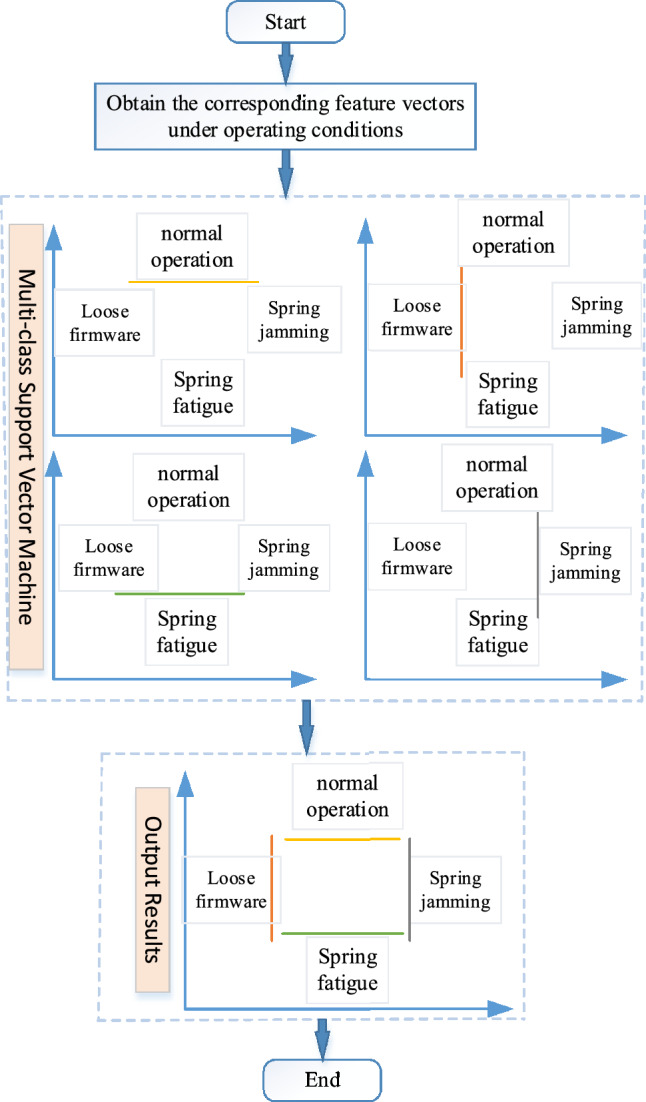
Schematic diagram of multi classification support vector machine results.

This article proposes an improved multi classification method based on the 1-v-1 support vector machine^25^, and applies the multi classification deep support vector machine to identify various types of faults. The structure diagram of the multi classification support vector machine is shown in Fig. 7.

From Fig. 7, the multi classification support vector machine establishes multiple hyperplanes, each hyperplane distinguishing a class of samples from the sample space, and ultimately completing the classification of multiple samples. The optimization problem is represented as follows^26,27^:

11 $$ \min \sum\limits_{d = 1}^{D} {\frac{1}{2}\theta_{d}^{\rm T} } \theta_{d} + e\sum\limits_{j = 1}^{J} {\sum\limits_{{d \ne f_{j} }} {\mu_{j}^{d} } } $$

12 $$ {\text{s}}.{\text{t}}. \;\;\;\;\;\theta_{{f_{j} }}^{\rm T} x_{j} + g_{{f_{j} }} \ge \theta_{d}^{\rm T} x_{j} + g_{d} + 2 - \mu_{j}^{d} $$

13 $$ \mu_{j}^{d} \ge 0,j \in [1,n],j \in N $$

where *θ*_*d*_ is the normal vector of the *d*-th classification hyperplane; ${g}_{{f}_{j}}$ is the bias term of the *f*_*j*_-th classification hyperplane; *f*_*j*_ is the classification label of the sample; *e* is the penalty factor; ${\mu }_{j}^{d}$ is the relaxation factor; *x*_*j*_ is the *j*-th sample; *D* is the number of hyperplanes for classification; *J* is the number of samples.

Based on the Lagrange theorem, establish the following function:

14 $$ \begin{gathered} L = \sum\limits_{d = 1}^{D} {\frac{1}{2}\theta_{d}^{\rm T} } \theta_{d} + e\sum\limits_{j = 1}^{J} {\sum\limits_{{d \ne f_{j} }} {\mu_{j}^{d} } } - \hfill \\ {\kern 1pt} {\kern 1pt} {\kern 1pt} {\kern 1pt} {\kern 1pt} {\kern 1pt} {\kern 1pt} {\kern 1pt} {\kern 1pt} {\kern 1pt} {\kern 1pt} {\kern 1pt} {\kern 1pt} {\kern 1pt} {\kern 1pt} \sum\limits_{j = 1}^{J} {\sum\limits_{d = 1}^{D} {\lambda_{j}^{d} \left[ {\left( {\theta_{{f_{j} }} - \theta_{d} } \right)^{T} x_{j} + g_{{f_{j} }} - g_{d} - 2 + \mu_{j}^{d} } \right]} } {\kern 1pt} - \sum\limits_{j = 1}^{J} {\sum\limits_{d}^{D} {\eta_{j}^{d} } } \mu_{j}^{d} \hfill \\ \end{gathered} $$

where $\lambda$, $\eta$ is the Lagrange factor, respectively。

Using the Lagrange method, find about *θ*_*d*_, *g*_*d*_, ${\mu }_{j}^{d}$ partial derivative of Eq. (14), then it can obtain:

15 $$ \left\{ {\begin{array}{*{20}c} {\frac{\partial L}{{\partial \theta_{d} }} = 0} \\ {\frac{\partial L}{{\partial g_{d} }} = 0} \\ {\frac{\partial L}{{\partial \mu_{j}^{d} }} = 0} \\ \end{array} } \right. $$

Substitute Eqs. (15) into (14) to obtain the dual optimization form:

16 $$ \max \;\sum\limits_{d = 1}^{D} {\sum\limits_{i = 1}^{J} {\sum\limits_{j = 1}^{J} {\left( {\lambda_{i}^{d} \lambda_{j}^{{f_{i} }} - \frac{1}{2}\lambda_{i}^{d} \lambda_{j}^{d} } \right)} } } x_{i}^{T} x_{j} + 2\sum\limits_{d = 1}^{D} {\sum\limits_{j = 1}^{J} {\lambda_{j}^{d} } } $$

$$ {\text{s}}.{\text{t}}.\sum\limits_{d = 1}^{D} {\sum\limits_{j = 1}^{J} {\lambda_{j}^{d} } } = \sum\limits_{j = 1}^{J} {q_{j}^{d} \lambda_{j} } ,d = {1},{2}, \ldots ,D $$

where $\lambda_{j}^{d}$ is Lagrange factor, with $\lambda_{j} = \sum\limits_{d = 1}^{D} {\lambda_{j}^{d} }$, $q_{j}^{k}$ is a parameter. And $q_{j}^{k}$ = 1, as *f*_*j*_ = *d*; $q_{j}^{k}$ = 0, as *f*_*j*_ ≠ *d*.

From this, the decision function of the multi classification support vector machine is obtained, represented as follows:

17 $$ p = {\text{sgn}} \mathop {\max }\limits_{d} \left[ {\sum\limits_{j = 1}^{J} {\left( {q_{j}^{d} K_{j} - \lambda_{j}^{d} } \right)x_{j}^{T} x_{j} + g_{d} } } \right] $$

where $$K_{j} = \sum\limits_{d = 1}^{D} {\lambda_{j}^{d} }$$.

The optimization problem of Eq. (11) is solved using the Lagrangian principle. The input of the multi classification support vector machine is the training sample set, and the output is the classification of the samples. The specific application process is as follows:

1. Using the singular value feature vector obtained from the previous text as input, train with Eq. (11) to establish a classification model;
2. Apply the Lagrange algorithm to obtain the dual form and obtain the decision function;
3. Determine the classification of each sample using the decision function of multi classification support vector machines.

### Application analysis of fault diagnosis for high voltage circuit breakers

**Figure 8:**
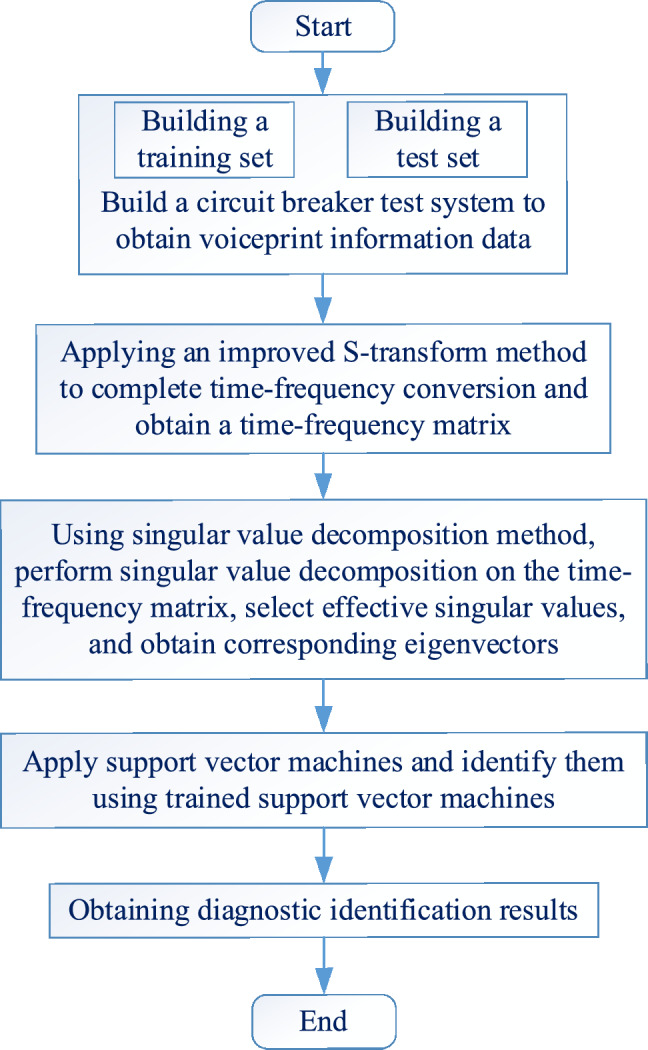
Circuit breaker fault identification and analysis process.

A comprehensive identification method for high-voltage circuit breaker faults is proposed through the improved S-transform, singular value decomposition, and support vector machine methods proposed in this article. The basic analysis flow chart of the proposed method is shown in Fig. 8.

According to the method proposed in this article, the detailed implementation steps for fault diagnosis are as follows.

- *Step 1* Build a testing system for the voiceprint information collection architecture of a certain type of high-voltage circuit breaker. On the testing system, set various operating conditions of the circuit breaker, and then collect voiceprint information data under different conditions. Randomly compile the voiceprint information data into training data sets and validation data sets.
- *Step 2* Using the improved S-transform proposed in the article, time–frequency conversion is performed on different types of training data sets to obtain corresponding feature matrices.
- *Step 3* Perform singular value decomposition on the feature matrix to obtain the corresponding eigenvectors.
- *Step 4* Input feature vectors into support vector machines for training.
- *Step 5* After completing step 2 and step 3, the voiceprint information data in the validation dataset is input into the trained support vector machine to obtain fault diagnosis results, and then the operation status monitoring of the circuit breaker is achieved.

## Experiments and analysis

### Establishment of experimental system and scenario analysis

**Figure 9:**
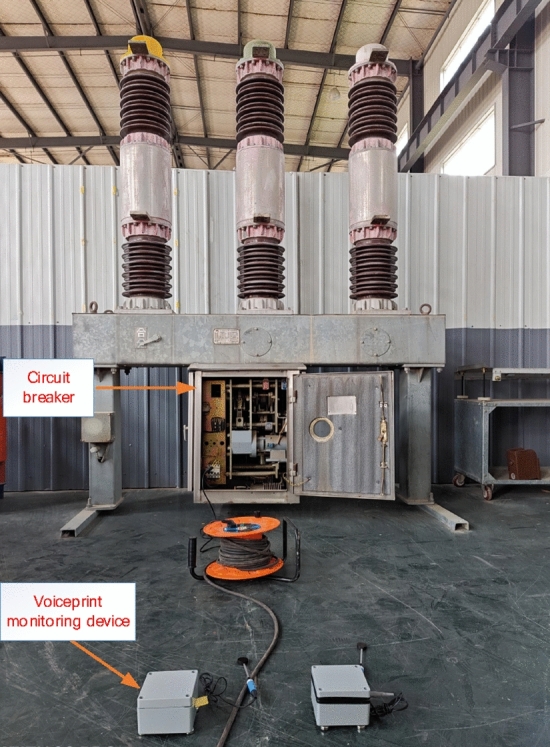
Voice print information monitoring test system.

Using the LW8-35A (T) circuit breaker as the tested circuit breaker, a test system is constructed as shown in Fig. 9. The test system mainly includes circuit breakers, voiceprint acquisition devices, auxiliary equipment, etc.

Manually control the opening and closing of the circuit breaker through the circuit breaker console. The sensors and software system of the monitoring device collect the voiceprint information of the circuit breaker, with a sampling frequency of 10 kHz and a collection time of 100 ms, and save it as a data file.

### Fault simulation

To accurately diagnose faults, it is necessary to establish a typical fault database for high-voltage circuit breakers. When applying the device, the trained model is used to classify and identify the collected data, in order to determine the type of fault. A typical fault database for basic circuit breakers has been established through preliminary research, and actual faults will be further simulated in a laboratory environment.

The main types of faults in high-voltage circuit breakers are mechanical faults. This test mainly simulated three types of mechanical faults, including: iron core jamming, loose components, and push and pull rod jamming, as shown in Table 1.

**Table 1:** Fault simulation type.

Number	Fault type	Fault simulation setting
1	Iron core jamming	Coil core hanging 50 g iron sheet
2	Loose components	The fixing nut of the opening and closing spring is loose by 5 mm
3	Pushing rod jamming	Hanging 1.2 kg iron sheet at the push and pull rod

**Figure 10:**
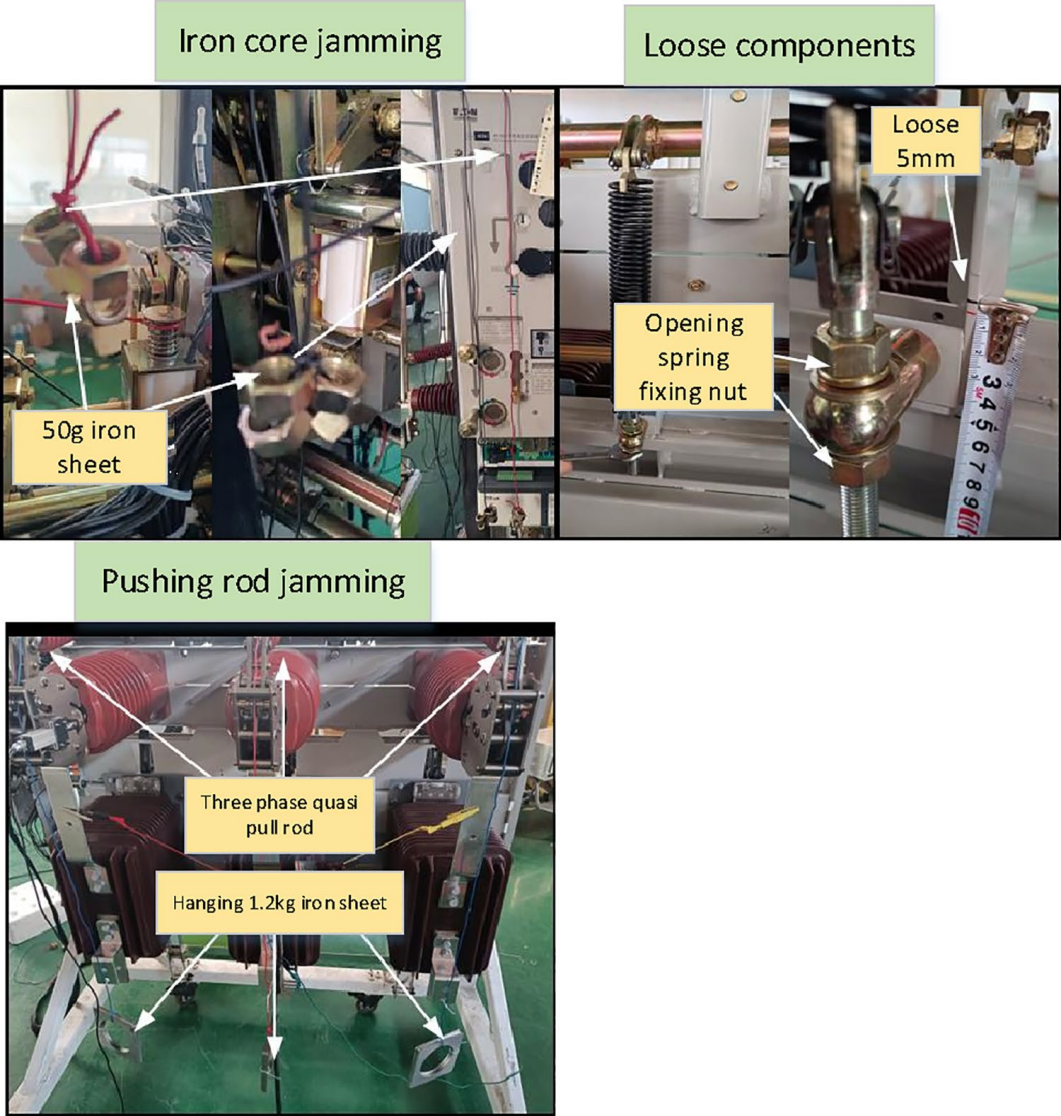
Fault simulation scenario.

According to the fault settings in Table 1, collect the voiceprint information data of circuit breaker opening and closing. Multiple fault simulation scenarios are shown in Fig. 10. At the same time, collect the voiceprint information under normal conditions, establish a database of voiceprint information.

### Diagnosis results analysis

**Figure 11:**
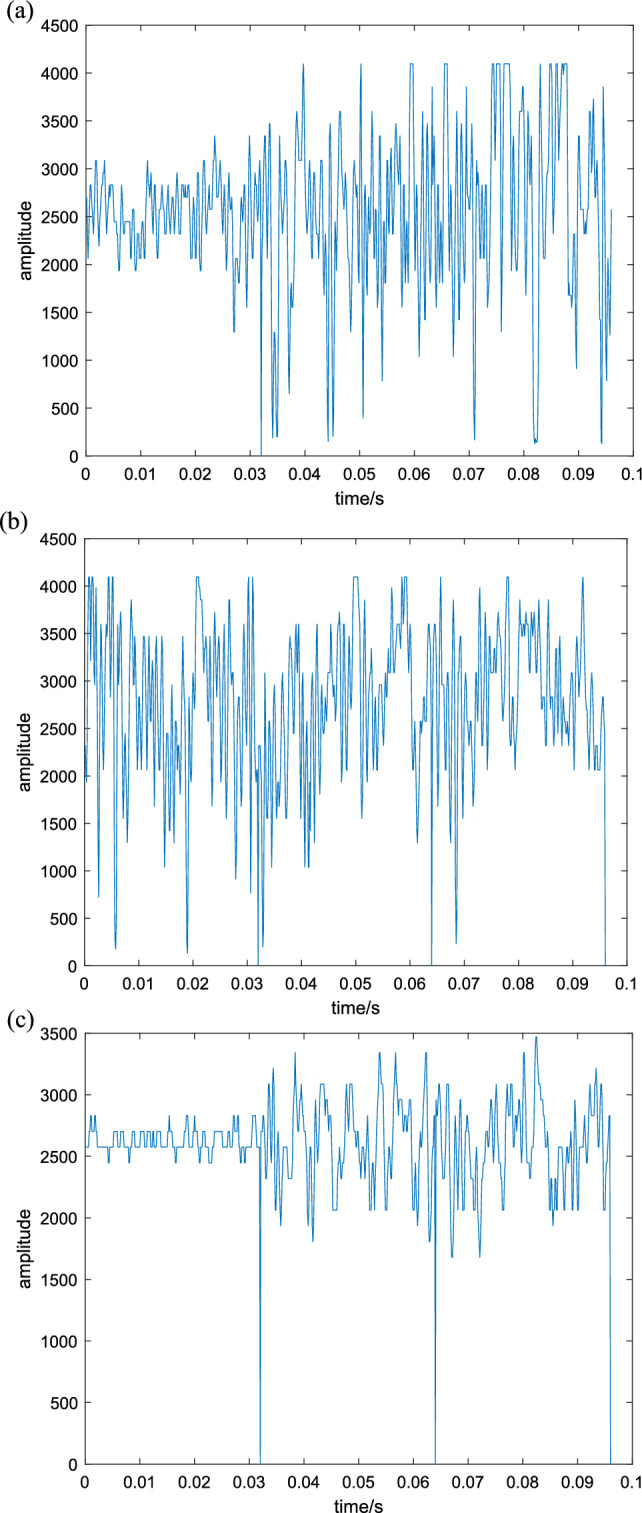
Typical state voiceprint information waveform of high-voltage circuit breakers. (**a**) Iron core jamming voiceprint information, (**b**) Pushing rod astringent voiceprint information, (**c**) Loose component voiceprint information.

Obtain the waveform of the voiceprint information data of the circuit breaker opening and closing under different working conditions from the established experimental system, as shown in Fig. 11.

Using the support vector machine algorithm, 15 samples were randomly selected for each working condition as the training dataset, and then 10 samples were selected as the test set. Under this training dataset and test set, the average prediction accuracy for each operating condition is obtained, as shown in Table 2.

**Table 2:** Diagnostic results under different working conditions of faults.

Number	Fault type	Accuracy of multi classification support vector machine method (%)	Accuracy of 1-v-1 support vector machine method (%)
1	Normal condition	94	91
2	Iron core jamming	87	86
3	Loose component	93	90
4	Pushing rod jamming	90	91
5	Overall	92.6	90.7

The calculation results in Table 2 indicate that the fault type diagnosis accuracy of the two different support vector machine methods is not the same. The multi classification support vector machine method presented in this article has improved accuracy and efficiency, and is also more convenient. By applying the calculation ideas and experimental system in this article, the overall accuracy of circuit breaker fault diagnosis has reached 92.6%, verifying the effectiveness of the monitoring device and experimental system.

In order to verify the performance of the comprehensive fault diagnosis method proposed in this article, a comparative calculation and analysis will be conducted with neural networks and decision trees. The diagnostic results will be calculated for each of the three fault types given in the article. This article evaluates the performance of different algorithms from two dimensions: accuracy of model algorithms and computation time.

The calculation results are shown in Table 3 below.

**Table 3:** Evaluation report on fault diagnosis using different algorithms.

Number	Fault type	The accuracy of the method in this article (%)	The accuracy of decision trees (%)	The accuracy of neural network (%)
1	Normal condition	94	90	89
2	Iron core jamming	87	80	79
3	Loose component	93	83	85
4	Pushing rod jamming	90	84	81
5	Overall	92.6	83.4	82.5

The calculation results in Table 3 indicate that the proposed comprehensive method has the best performance. The overall diagnostic accuracy of the decision tree is 83.4%, while the accuracy of the neural network is 82.5%. The worst accuracy of decision trees is 80%, and the worst accuracy of neural networks is 81%.

The average calculation time for diagnosing three types of faults using the method proposed in this article is 36.7 s, the average calculation time for decision trees is 43.5 s, and the average calculation time for neural networks is 41.9 s.

Based on the above analysis, the comprehensive diagnostic method proposed in this article has the best performance.

The calculation results under various operating conditions indicate that the comprehensive identification method proposed in this paper can effectively diagnose circuit breaker faults, verifying the feasibility and adaptability of the equipment and the proposed technical analysis method.

## Conclusions

This article develops a voiceprint monitoring device for high-voltage circuit breakers. Based on voiceprint information data, a mechanical fault diagnosis method for high-voltage circuit breakers is proposed. The conclusions are as follows:

1. The time–frequency domain conversion and feature extraction of the opening and closing voiceprint information of high-voltage circuit breakers under different working conditions are carried out to form the original feature space, which fully describes the voiceprint characteristics.
2. The developed high voltage circuit breaker voiceprint information acquisition device is easy to use and has strong functionality. The built high voltage circuit breaker simulation mechanical fault diagnosis system has good adaptability and reliability, laying a certain foundation for future promotion and application.
3. The proposed fault comprehensive diagnosis method consisting of improved S-transform, singular value decomposition, and multi classification support vector machine can effectively extract features from the collected voiceprint information data and reliably complete fault classification diagnosis. The experimental results show that the comprehensive method has high identification accuracy and efficiency.
4. The test results indicate that the developed high-voltage circuit breaker voiceprint monitoring equipment and simulation test system have good application feasibility, and fault diagnosis has shown good results, providing new ideas for intelligent operation and maintenance of substations.

In future research, comprehensive fault diagnosis techniques for mechanical and non mechanical faults of high-voltage circuit breakers will be studied by obtaining voiceprint information and non voiceprint information data of circuit breakers.

## Data Availability

The data that support the findings of this study are available from the corresponding authors upon reasonable request.
